# Predictors of food insecurity among older adults before and during COVID-19 in the United States

**DOI:** 10.3389/fpubh.2023.1112575

**Published:** 2023-05-12

**Authors:** Emily Joy Nicklett, Greta Jianjia Cheng, Zachary A. Morris

**Affiliations:** ^1^Department of Social Work, College for Health, Community and Policy, University of Texas at San Antonio, San Antonio, TX, United States; ^2^Department of Epidemiology, School of Public Health, University of Pittsburgh, Pittsburgh, PA, United States; ^3^School of Social Welfare, Stony Brook University, Stony Brook, NY, United States

**Keywords:** food insecurity, food security, COVID-19, older adults, disability, Health and Retirement Study

## Abstract

**Background:**

The COVID-19 pandemic has strained the health and wellbeing of older adult populations through increased morbidity, mortality, and social exclusion. However, the impact of COVID-19 on the health of older adults through food security has received relatively little attention, despite the strong impact of diet quality on the health and longevity of older adults.

**Objective:**

The objective of this study was to identify sociodemographic and socioeconomic predictors of self-reported food insecurity before and early in the COVID-19 pandemic among community-dwelling older adults in the United States.

**Methods:**

Using longitudinal data from the Health and Retirement Study, a nationally representative sample of middle-aged and older adults in the United States, we examined the associations between sociodemographic and socioeconomic predictors of self-reported food insecurity between 2018 (*N* = 2,413) and June 2020 (*N* = 2,216) using population-weighted multivariate logistic regression models.

**Results:**

The prevalence of food insecurity doubled among participants from 2018 (4.83%) to June 2020 (9.54%). In 2018, non-Hispanic Black and rural residents were more likely to report food insecurity, while individuals with higher education and greater wealth were less likely to report food insecurity in adjusted models. In June 2020, those who were relatively younger, not working due to a disability, and renting were more likely to report food insecurity. Those with an increased number of functional limitations, a recent onset of a work-limiting disability, and those who were no longer homeowners experienced an elevated longitudinal risk for food insecurity.

**Conclusion:**

Future research should examine effective policies and interventions to address the disproportionate impacts of COVID-19 on populations at a heightened risk of experiencing food insecurity.

## 1. Introduction

The COVID-19 pandemic has particularly strained the health and wellbeing of older adult populations through increased morbidity, mortality, and social exclusion. However, the impact of COVID-19 on the health of older adults through food access and food security has received relatively little attention, despite the strong impact of diet quality on the health and longevity of older adult populations (1–4). Food insecurity is defined as having limited or uncertain access to adequate nutritious food to maintain an active and healthy life (5). Studies of the early food insecurity impacts of COVID-19 have found as much as a one-third increase (32.3%) in household food insecurity overall since the onset of COVID-19, with 35.5% of food insecure households classified as a newly food insecure in the United States (6).

During the COVID-19 pandemic, food security has been affected by stay-at-home orders, closure/limited hours of food retailers, supply chain issues, the relatively sudden surge of high unemployment, inflation, and other economic impacts. In particular, the pandemic has highlighted challenges in food access and food security for older adults. Older adults could be disproportionately affected due to increased financial hardship, reduced use of public transportation, and less access to food delivery services among this population. Because of older adults' relatively high vulnerability to morbidity and mortality from COVID-19, older adults might be concerned about their safety while accessing grocery retailers (7). Social distancing policies may also hinder older adults' ability to benefit from community food resources, such as the Supplemental Nutrition Assistance Program (SNAP) and food banks. This is likely particularly true for older adults with disabilities who are disproportionately affected by COVID-19 (8, 9).

Food insecurity is a chronic, longstanding issue that has been exacerbated during COVID-19. Historically, food insecurity has disproportionately impacted people of color and low-income households, mainly because communities of color and low-income communities are less likely to have geographically and economically accessible healthy food than predominantly white communities and more affluent communities (10–12). Among middle-aged and older populations, women and those of relatively younger age were found to be more susceptible to food insecurity (13, 14). Previous studies have also linked food insecurity in older adults to multiple chronic conditions (13, 15, 16) and functional limitations (15, 17, 18). The presence of chronic illness comorbidities and functional limitations may adversely affect individuals' ability to shop for food, carry food home, and prepare meals, potentially contributing to food insecure conditions.

Persistent gender, socioeconomic, racial, and ethnic disparities in food insecurity during COVID-19 have been consistently observed across all age groups (6, 19–25). While some studies have examined the differential impacts of COVID-19 on food security and food access using longitudinal data to examine changes in food insecurity before and after the onset of the pandemic (26, 27), no studies have focused on risk and protective factors specific to older adult populations (28). Little is known about the risk and protective factors associated with food insecurity among diverse middle-aged and older adult populations. Using a nationally representative sample of adults aged 50 and older in the United States, the present study examines sociodemographic and socioeconomic predictors of food insecurity before COVID-19 (2018) and during COVID-19 (since June 2020). We also investigate time-varying longitudinal risk factors for food insecurity during the pandemic. Sociodemographic characteristics examined include age, gender, race/ethnicity, partnership status, and urbanicity. Socioeconomic characteristics examined include educational attainment, total household wealth, individual income, current working status, and home ownership status.

## 2. Methods

### 2.1. Sample

The data utilized in this study were from a longitudinal cohort of older adults who completed the June COVID module (2020) and 2018 waves of the Health and Retirement of Study (HRS). HRS is a nationally representative longitudinal survey of adults over the age of 50 in the United States that began in 1992 and continued with data collection every 2 years since. A multi-stage probability sampling strategy was utilized by HRS with an adjustment for geographic stratification, clustering, and oversampling of African Americans and Hispanic/Latinx populations (29). In 2020, HRS added COVID-19-specific questions to the core interview which were fielded to a 50% random subsample of households. Questionnaires were administrated to one-half of the subsample on 11 June 2020 and to the other half of the subsample on 24 September 2020. The current study used data from the June 2020 release, which includes 3,266 respondents, accounting for a random sample of approximately 25% of HRS participants. Pre-pandemic data for the same 3,266 respondents were drawn from 2018 HRS survey data.

Of the 3,266 respondents in 2018, 853 (26%) were excluded from the analysis on predictors of 2018 food security status for the following reasons: (1) younger than 50 years of age (*n* = 96); (2) missing food security status (*n* = 62); (3) missing observations for any independent variables (*n* = 493); and (4) missing sampling weight (*n* = 304). The analytical sample for 2018 is 2,413. Of those 3,266 respondents in June 2020, 1,050 (32%) were excluded from the analysis on predictors of 2020 food security status for the following reasons: (1) younger than 50 years of age (*n* = 96); (2) missing food security status (*n* = 853); (3) missing observations for any independent variables (*n* = 76); and (4) missing sampling weight (*n* = 25). The analytical sample for 2020 is 2,216.

The HRS was approved by the University of Michigan Health Sciences/Behavioral Sciences Institutional Review Board (HUM00061128). The core HRS questionnaires for 2018 and 2020 can be accessed at https://hrsdata.isr.umich.edu/data-products/public-survey-data?_ga=2.76255637.1075731333.1676136739-821463064.1675792997.

### 2.2. Measures

#### 2.2.1. Food insecurity

Food insecurity was identified through two self-reported measures as developed in prior research (30, 31). Participants were asked whether, since the last interview, they always had enough money to buy the food they needed. Response options were coded as *yes, no, don't know*, or *refused*. Participants who answered “no” to the first question were then asked whether, in the past 12 months, they ever ate less than they felt they should because there was not enough money to buy food. Response options were coded as *yes, no, don't know*, or *refused*. These two questions were used to create a dichotomous variable to categorize food security status in 2018 and 2020 (30, 31). Participants reporting that they had enough money to buy the food they needed since the last interview were considered food secure. Participants reporting that they did not have enough money to buy the food they needed since the last interview and ate less in the past 12 months were considered as food insecure. This self-reported measure is consistent with prior research using the HRS (30, 31), in line with conceptual developments in food security measurement toward the use of subjective measures (32), and consistent with other widely used self-reported measures of food insecurity, including the measure adopted by the United States Department of Agriculture (USDA) (33).

#### 2.2.2. Sociodemographic characteristics

Sociodemographic characteristics examined in this study included age group (50–64, 65–74, and 75+), gender (male and female), partnership status (uncoupled and coupled), race/ethnicity (non-Hispanic White, non-Hispanic Black, Hispanic/Latinx, and non-Hispanic Other), and urbanicity (urban, suburban, and ex-urban), which was classified by following the 2013 Beale Rural-Urban Continuum Codes. Age group and partnership status were measured in 2018 and 2020, while gender, race/ethnicity, and urbanicity were only assessed at baseline (2018).

#### 2.2.3. Socioeconomic characteristics

We included socioeconomic characteristics that are associated with food access, diet quality, and food insecurity among older adults in recent studies (28, 34, 35). Examined socioeconomic characteristics include educational attainment (less than high school, high school or General Education Diploma (GED) completion, some college, and college or more), total wealth (negative net wealth, below median, and above median), individual income (no income, below median, and above median), current working status (currently working, not currently working, not working due to disability, retired, and others), and home ownership status (own, rent, and other). Total wealth (including secondary residence) was calculated by total assets minus total debts.^1^ The individual income included the respondent's total earnings from salaries, wages, bonuses received from employment and self-employment, and investments. Total wealth, current working status, and home ownership status were assessed in 2018 and 2020, while only baseline measures were available for educational attainment and individual income.

#### 2.2.4. Health-related characteristics

We controlled for health-related characteristics that may confound the associations between food security status and the sociodemographic and socioeconomic predictors. Functional limitations were assessed by instrumental activities of daily living (IADLs). IADLs were measured by the level of assistance needed to use a telephone, take medication, and handle money (36). Participants were assessed whether they were able to complete each IADL item without assistance (0) or with assistance (1). The final scores were summed for IADLs (range: 0–3) to indicate functional limitations. The number of chronic illness comorbidities was assessed using participants' reports of diagnosis with eight potential conditions—including arthritis, cancer, diabetes, heart disease, hypertension, lung disease, psychiatric problems, and stroke (range: 0–8). Chronic illness comorbidities were assessed at baseline (2018) only, while the measure of IADLs was time-varying.

### 2.3. Analytic approach

We characterized food security status in the unweighted sample in 2018 and 2020. Parametric *t*-tests and chi-squared tests were conducted to assess associations between sample characteristics and food security status in 2018 and 2020. We then examined the independent relationships between sociodemographic and socioeconomic characteristics and food insecurity in 2018 and 2020 using two separate binomial logistic regression models.^2^ Odds ratios and the corresponding 95% confidence intervals were reported to compare the relative odds of food insecurity across sociodemographic/socioeconomic subgroups. Functional limitations and chronic disease comorbidities were included as control variables, as these characteristics could confound the relationship between sociodemographic and socioeconomic characteristics and food insecurity among older adults (13, 15–18, 37). We further exploit the longitudinal nature of the data by coding the available time-varying measures according to changes observed from 2018 to 2020 (38). For example, in the case when a respondent reported a work disability in 2020 but not in 2018, we generate a variable indicating a “new disability onset” which is compared with those with no change in disability status. We then examined the relationship between the time-varying variables (IADLs, working status, partnership status, and homeownership status) and the onset of food insecurity in 2020 to identify those at risk for food insecurity during the pandemic. All regression analyses were weighted^3^ to adjust for selection and non-response biases. Multicollinearity was not a concern, as variance inflation factors (VIFs) for all predictor variables were below 1.50, well below the established threshold of 4.0 (39). All analyses were conducted using Stata 17.0 SE (College Station, TX).

## 3. Results

The mean age of the sample was 66.9 years (SD: 10.3, range: 50–99) at baseline. As shown in Table 1, all examined sociodemographic, socioeconomic, and health-related characteristics are significantly associated with food insecurity (*p* < 0.05) in 2018 and/or 2020, except for urbanicity and income. See Table 1 for complete sample characteristics and bivariate analyses (unweighted).

**Table 1:** Unweighted sample characteristics in 2018 and 2020 by food security status, Health and Retirement Study.

	**Total sample 2018 *n =* 2,413**	**Food insecure 2018 *n =* 90**	**Food secure 2018 *n =* 2,323**		**Total sample 2020 *n =* 2,216**	**Food insecure 2020 *n =* 211**	**Food secure 2020 *n =* 2,005**	
	**M(SD)/% (** ***n*** **)**	**M(SD)/% (** ***n*** **)**	**M(SD)/% (** ***n*** **)**	***p*** **-value**	**M(SD)/% (** ***n*** **)**	**M(SD)/% (** ***n*** **)**	**M(SD)/% (** ***n*** **)**	***p*** **-value**
**Age group**				*p =* 0.33				*p* < 0.0001
50–64	49.0 (1,183)	53.3 (48)	48.9 (1,135)		38.8 (859)	62.6 (132)	36.3 (727)	
65–74	25.6 (617)	18.9 (17)	25.8 (600)		28.9 (640)	26.1 (55)	29.2 (585)	
75+	25.4 (613)	27.8 (25)	25.3 (588)		32.4 (717)	11.4 (24)	34.6 (693)	
**Gender** ^a^				*p =* 0.03				*p =* 0.02
Male	37.5 (905)	26.7 (24)	37.9 (811)		36.9 (817)	29.4 (62)	37.7 (755)	
Female	62.5 (1,508)	73.3 (66)	62.1 (1,442)		63.1 (1,399)	70.6 (149)	62.3 (1,250)	
**Race/ethnicity** ^a^				*p* < 0.0001				*p =* 0.69
Non-Hispanic White	77.8 (1,876)	50.0 (45)	78.8 (1,831)		74.2 (1,644)	73.0 (154)	74.3 (1,490)	
Non-Hispanic Black	14.3 (346)	36.7 (33)	13.5 (313)		16.0 (355)	18.0 (38)	15.8 (317)	
Hispanic/Latinx	5.7 (137)	10.0 (9)	5.5 (128)		7.5 (166)	6.2 (13)	7.6 (153)	
Non-Hispanic Other	2.2 (54)	3.3 (3)	2.2 (51)		2.3 (51)	2.8 (6)	2.2 (45)	
**Partnership status**				*p =* 0.001				*p =* 0.004
Uncoupled	45.0 (1,086)	62.2 (56)	44.3 (1,030)		57.4 (1,272)	66.8 (141)	56.4 (1,131)	
Coupled	55.0 (1,327)	37.8 (34)	55.7 (1,293)		42.6 (944)	33.2 (70)	43.6 (874)	
**Urbanicity** ^a^				*p =* 0.12				*p* < 0.09
Urban	48.3 (1,166)	40.0 (36)	48.6 (1,130)		47.6 (1,054)	45.4 (96)	47.8 (958)	
Suburban	24.2 (585)	23.3 (21)	24.3 (564)		24.4 (541)	20.4 (43)	24.8 (498)	
Ex-urban/ rural	27.4 (662)	36.7 (33)	27.1 (629)		28.0 (621)	34.1 (72)	27.4 (549)	
**Education** ^a^				*p* < 0.0001				*p =* 0.79
Less than high school	18.6 (585)	38.7 (58)	17.7 (523)		18.6 (585)	16.6 (35)	18.8 (376)	
High school/ GED	37.1 (1,168)	40.7 (61)	37.1 (1,097)		37.1 (1,168)	39.3 (83)	36.8 (737)	
Some college	22.2 (709)	15.3 (23)	22.9 (676)		22.2 (709)	23.2 (49)	22.3 (446)	
College or more	21.79 (686)	5.33 (8)	22.35 (661)		21.79 (686)	20.9 (44)	22.2 (445)	
**Total wealth**				*p* < 0.0001				*p =* 0.82
Negative net wealth	3.7 (89)	16.7 (15)	3.2 (74)		4.1 (87)	3.9 (8)	4.2 (79)	
Below median	45.3 (1,092)	67.8 (61)	44.4 (1,031)		47.7 (1,002)	50.0 (101)	47.4 (901)	
Above median	51.1 (1,232)	15.6 (14)	52.4 (1,218)		48.2 (1,014)	46.3 (94)	48.4 (920)	
**Individual income** ^a^				*p =* 0.23				*p =* 0.31
No income	90.0 (2,171)	94.4 (85)	89.8 (2,086)		89.9 (1,992)	92.9 (196)	89.6 (1,796)	
Below median	5.3 (128)	4.4 (4)	5.3 (124)		5.2 (116)	3.8 (8)	5.4 (108)	
Above median	4.7 (114)	1.0 (1)	4.9 (113)		4.9 (108)	3.3 (7)	5.0 (101)	
**Current working status**				*p* < 0.0001				*p* < 0.0001
Currently working	8.1 (257)	4.4 (4)	8.2 (191)		26.4 (585)	23.2 (49)	26.7 (536)	
Not currently working	6.1 (147)	3.3 (3)	6.2 (144)		9.5 (210)	10.0 (21)	9.4 (189)	
Not working due to disability	4.4 (106)	17.8 (16)	3.9 (90)		12.9 (285)	38.9 (82)	10.1 (203)	
Retired	81.4 (1,965)	74.4 (67)	81.7 (2,343)		49.2 (1,091)	23.7 (50)	51.9 (1,041)	
Others	0 (0)	0 (0)	0 (0)		2.0 (45)	4.3 (9)	1.8 (36)	
**Home ownership status**				*p =* 0.001				*p* < 0.0001
Owning a home	83.5 (2,015)	71.1 (64)	84.0 (1,951)		67.3 (1,491)	43.6 (92)	69.8 (1,399)	
Renting	12.2 (295)	24.2 (22)	11.8 (273)		27.8 (617)	48.8 (103)	25.6 (514)	
Others	4.3 (103)	4.4 (4)	4.3 (99)		4.9 (108)	7.6 (16)	4.5 (92)	
**IADL limitations (0–3)**	0.2 (0.60)	0.4 (0.79)	0.2 (0.59)	*p =* 0.45	0.4 (0.48)	0.3 (0.67)	0.1 (0.46)	*p* < 0.0001
**Chronic illness count** ^a^	2.4 (0.85)	2.7 (0.65)	2.4 (0.86)	*p =* 0.001	2.4 (0.86)	2.5 (0.84)	2.4 (0.86)	*p =* 0.13

a Only 2018 data are available.

Table 2 reports the population-weighted multivariate logistic regression models. The prevalence of food insecurity nearly doubled from 2018 (4.83%) to 2020 (9.54%) in unweighted, unadjusted models.

**Table 2:** Population-weighted multivariate logistic regression predicting sociodemographic and socioeconomic correlates of food insecurity in 2018 and 2020, Health and Retirement Study.

	**2018 *n =* 2,413**		**2020 *n =* 2,216**	
	**OR (95% CI)**	***p*** **-value**	**OR (95% CI)**	***p*** **-value**
**Age group (ref: 75**+**)**				
50–64	0.81 (0.45, 1.49)	0.504	5.23 (2.44, 11.21)	<0.0001
65–74	0.61 (0.30, 1.24)	0.173	4.80 (2.33, 9.91)	<0.0001
**Female** ^a^	1.32	0.305	1.57	0.051
**(ref: Male)**	(0.77, 2.26)		(1.00, 2.48)	
**Race/ethnicity**^a^ **(ref: non-Hispanic White)**				
Non-Hispanic Black	2.43 (1.41, 4.19)	0.001	1.09 (0.59, 2.01)	0.777
Hispanic/Latinx	1.49 (0.64, 3.50)	0.357	0.98 (0.41, 2.35)	0.963
Non-Hispanic Other	2.36 (0.59, 9.40)	0.222	1.80 (0.43, 7.54)	0.419
**Coupled**	0.82	0.444	0.69	0.14
**(ref: uncoupled)**	(0.50, 1.35)		(0.43, 1.13)	
**Urbanicity**^a^ **(ref: urban)**				
Suburban	1.19 (0.66, 2.14)	0.562	0.70 (0.40, 1.25)	0.231
Ex-urban	1.81 (1.04, 3.15)	0.036	1.00 (0.61, 1.63)	0.992
**Education**^a^ **(ref: less than high school)**				
High school/GED	0.73 (0.39, 1.34)	0.309	1.19 (0.66, 2.16)	0.568
Some college	0.49 (0.22, 1.07)	0.074	0.88 (0.45, 1.71)	0.699
College or more	0.27 (0.09, 0.79)	0.017	1.10 (0.57, 2.13)	0.777
**Total wealth (ref: negative net wealth)**				
Below median	0.34 (0.15, 0.75)	0.007	0.97 (0.54, 1.72)	0.906
Above median	0.12 (0.05, 0.33)	<0.0001	1.11 (0.66, 1.85)	0.699
**Individual income**^a^ **(ref: no income)**				
Below median	1.38 (0.44, 4.26)	0.579	0.98 (0.38, 2.58)	0.971
Above median	0.21 (0.02, 2.25)	0.197	0.51 (0.20, 1.30)	0.159
**Current working status (ref: currently working)**				
Not currently working	0.53 (0.09, 2.99)	0.474	1.33 (0.61, 2.90)	0.472
Not working due to a disability	2.64 (0.60, 11.70)	0.2	3.10 (1.72, 5.58)	<0.0001
Retired	1.16 (0.31, 4.34)	0.822	0.86 (0.42, 1.76)	0.679
Others	–	–	2.26 (0.61, 8.26)	0.221
**Home ownership status (ref: homeowner)**				
Renting	0.73 (0.38, 1.43)	0.362	2.96 (1.85, 4.74)	<0.0001
Others	0.60 (0.22, 1.65)	0.322	1.49 (0.54, 4.10)	0.436
**IADL limitations**	1.07	0.661	2.00	<0.0001
**(0–3)**	(0.78, 1.47)		(1.46, 2.75)	
**Chronic disease**	1.41	0.071	0.88	0.354
**count** ^a^	(0.97, 2.05)		(0.69, 1.15)	

a Only 2018 data are available. “–” estimates were omitted due to the insufficient sample size.−2 Log-likelihood results: 2018 = 698.82 (*p* < 0.0001); 2020 = 1,201.14 (*p* < 0.0001).

**Figure 1:**
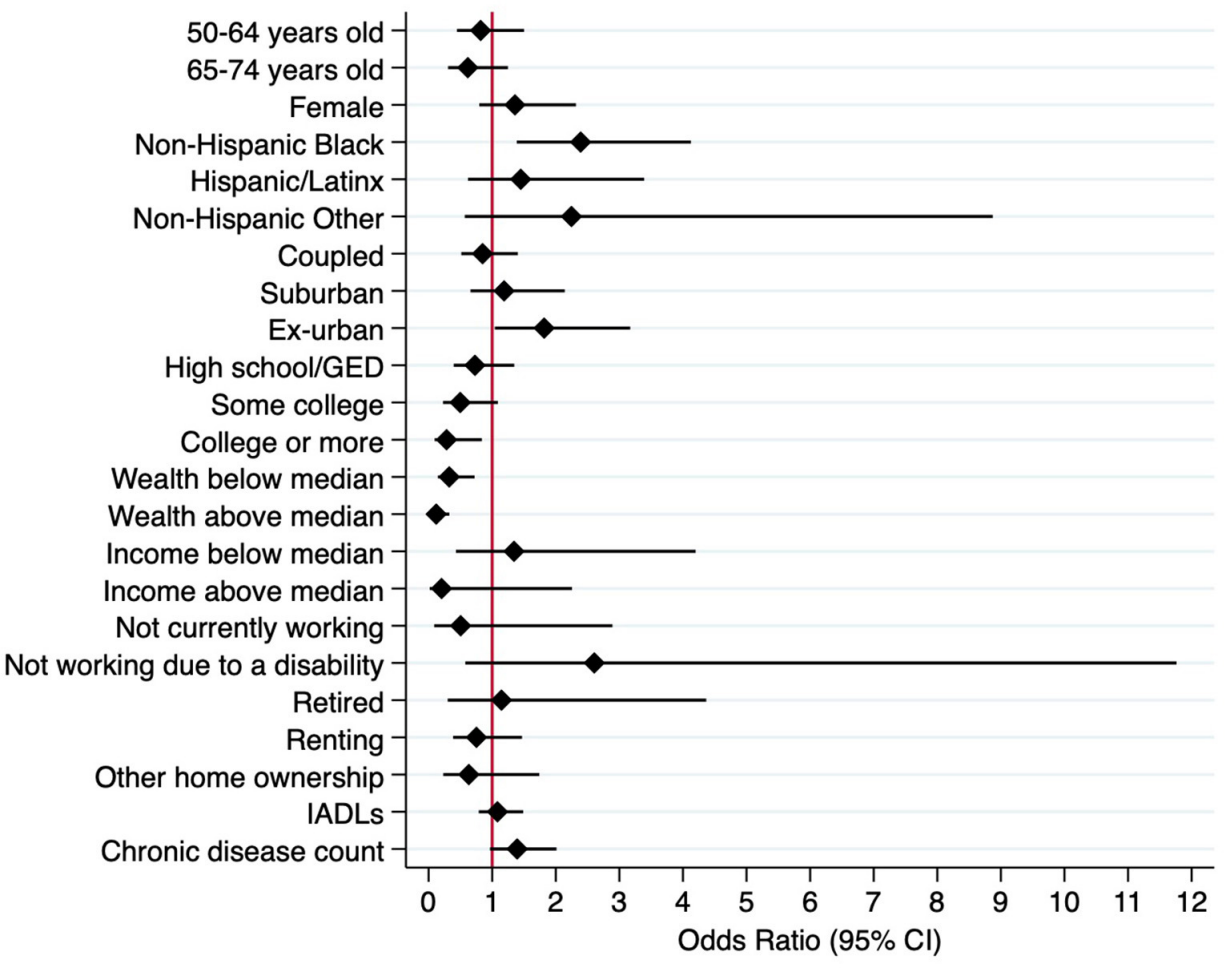
Weighted multivariate logistic regression model predicting food insecurity, 2018 (*N* = 2,413).

According to these population-weighted multivariate models, in 2018 specifically (Table 2; Figure 1), significant sociodemographic correlates of food insecurity included race/ethnicity and urbanicity.

Non-Hispanic Blacks had 2.43 times (95% CI: 1.41, 4.19) higher odds of experiencing food insecurity than non-Hispanic Whites. In contrast to urban residents, rural/ex-urban residents had 1.81 times (95% CI: 1.04, 3.15) higher odds of experiencing food insecurity. Age, gender, and partnership status were not found to be associated with food security status in 2018. In 2018, significant socioeconomic correlates of food insecurity included educational attainment and wealth. Individuals with college-level education or more had 0.27 times (95% CI: 0.09, 0.79) lower odds of reporting food insecurity than individuals with less than a high school education. Relative to individuals with negative net wealth, individuals with wealth value below the median experienced 0.34 times (95% CI: 0.15, 0.75) lower odds of food insecurity. Similarly, individuals with wealth value above the median experienced 0.12 times (95% CI: 0.05, 0.33) lower odds of food insecurity. Individual income, current working status, and home ownership status were not associated with food insecurity status in 2018.

**Figure 2:**
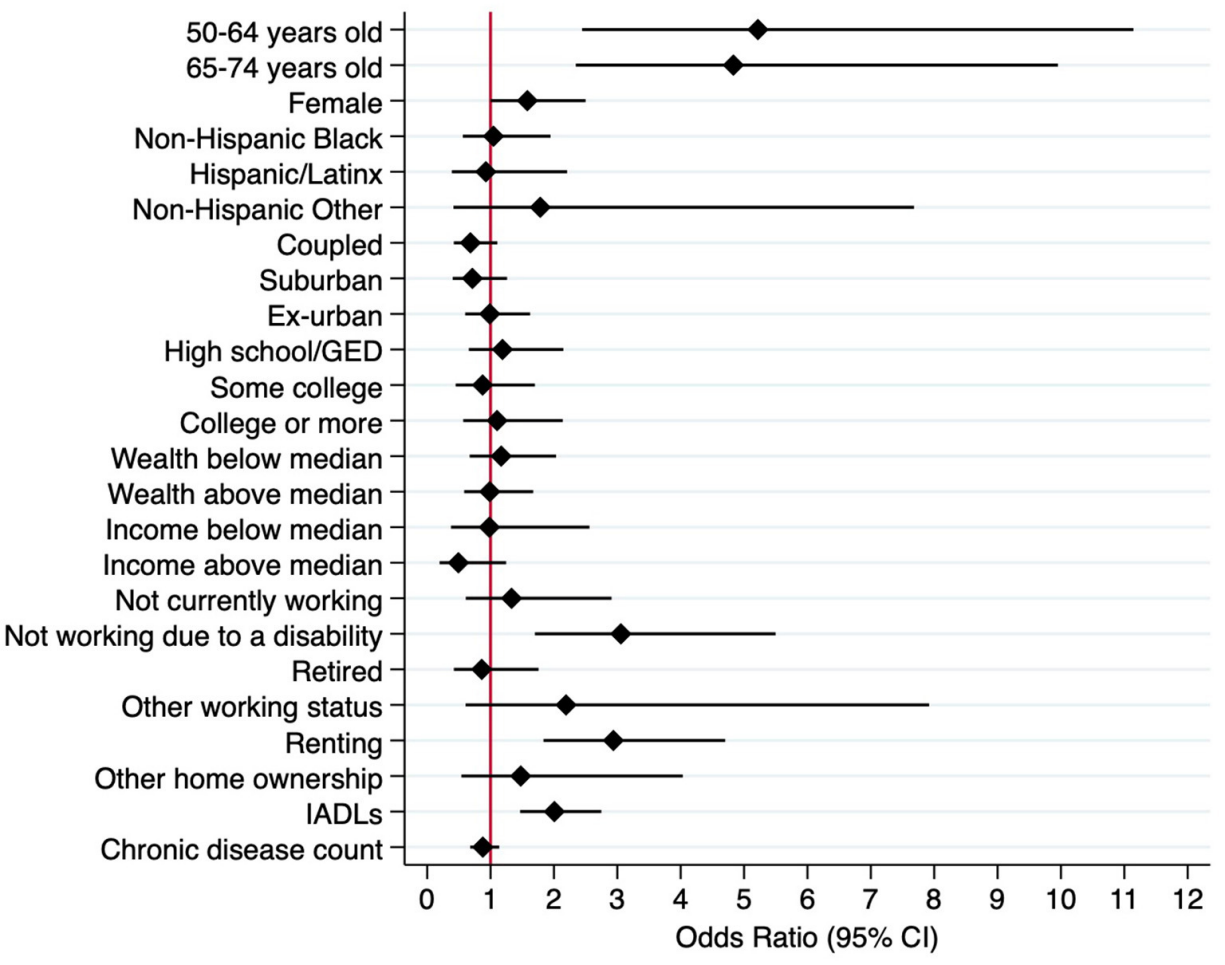
Weighted multivariate logistic regression model predicting food insecurity, 2020 (*N* = 2,216).

In June 2020 (Table 2; Figure 2), early in the pandemic, relatively younger age was a significant sociodemographic predictor of food insecurity. Those aged 50–64 (vs. 75+) had 5.23 times (95% CI: 2.44, 11.21) higher odds of food insecurity, and those aged 65–74 (vs. 75+) had 4.80 times (95% CI: 2.33, 9.91) higher odds of experiencing food insecurity. Other examined sociodemographic characteristics—including race/ethnicity, partnership status, and urbanicity—were not associated with the food security status in 2020. Early in the pandemic (June 2020), significant socioeconomic correlates of food insecurity included current working status and home ownership status. Respondents who were not able to work due to a disability experienced 3.10 times (95% CI: 1.72, 5.58) higher odds of food insecurity than those currently working. In comparison with respondents who owned their homes, those who were renting experienced 2.96 times (95% CI: 1.85, 4.74) higher odds of food insecurity. Those with greater IADL limitations experienced 2.00 times (95% CI: 1.46, 2.75) higher odds of food insecurity. Educational attainment, wealth, and individual income were not associated with food security status in 2020.

In Table 3, we apply logistic regression to examine longitudinal predictors of the onset of food insecurity in 2020 when controlling for baseline food insecurity (model 1) and when restricted to those who did not experience food insecurity in 2018 (model 2). The results from model 1, which are similar to those in model 2, indicate that those who developed a greater number of IADL limitations from 2018 to 2020 experienced 4.82 times (95% CI: 2.56, 9.07) higher odds of food insecurity in 2020. Those with a reduced number of IADLs in 2020 also experienced 1.96 times (95% CI: 0.96, 4.00) higher odds for food insecurity relative to those with no change in their IADLs, although it is notable that this risk is lower than for those whose number of functional limitations increased. Recent onset of a work disability was also associated with 2.35 times (95% CI: 1.32, 4.15) higher odds of experiencing food insecurity relative to no change in work disability status. Those who were homeowners in 2018 but were no longer homeowners in 2020 experienced 2.75 (95% CI: 1.66, 4.55) times higher odds of food insecurity relative to those whose homeownership status did not change.

**Table 3:** Longitudinal predictors of food insecurity in 2020, population-weighted, Health and Retirement Study^a^.

		**Model controlling for food insecurity in 2018 *n* = 2,086**		**Model excluding those with food insecurity in 2018 *n* = 1,981**	
	**n**	**OR (95% CI)**	***p*** **-value**	**OR (95% CI)**	***p*** **-value**
IADLs worsen	256	4.82 (2.56,9.07)	0.000	4.89 (2.56,9.34)	0.000
IADLs improve	420	1.96 (0.96,4.00)	0.064	2.04 (0.95,4.40)	0.069
Work-disability onset	362	2.35 (1.32,4.15)	0.003	2.13 (1.18,3.83)	0.012
No longer report work-disability	168	1.20 (0.33,4.37)	0.780	1.37 (0.37,5.06)	0.637
No longer working	197	0.81 (0.28,2.32)	0.694	0.70 (0.21,2.29)	0.554
Newly retired	331	0.52 (0.17,1.62)	0.259	0.53 (0.16,1.74)	0.296
Back to work after retired	684	0.76 (0.41,1.41)	0.384	0.66 (0.34,1.26)	0.206
Continuously working	60	0.30 (0.07,1.29)	0.105	0.27 (0.06,1.15)	0.077
No longer coupled	733	1.00 (0.59,1.68)	0.987	1.03 (0.60,1.76)	0.914
Newly coupled	841	0.90 (0.49,1.64)	0.723	0.90 (0.48,1.67)	0.730
No longer homeowner	476	2.75 (1.66,4.55)	0.000	2.71 (1.61,4.54)	0.000
New homeowner	363	0.76 (0.33,1.77)	0.529	0.71 (0.28,1.79)	0.464
Food insecurity (2018)	150	1.45 (0.55,3.83)	0.450	—	—

Both models control for age, gender, race, urbanicity, income, education, wealth, and the number of health conditions in 2018.

## 4. Discussion

To the best of our knowledge, the current study is among the first to examine whether selected sociodemographic and socioeconomic characteristics were associated with food insecurity before the COVID-19 pandemic (2018) and in the early pandemic (June 2020) among a nationally representative sample of community-dwelling middle-aged and older adults in the United States. Using a retrospective cohort, we were able to capture individual food security status before the COVID-19 pandemic and compare the changes in sociodemographic and socioeconomic correlates of food security before and early in the pandemic. We further examined longitudinal risk factors for the onset of food insecurity during the pandemic, which represents an advancement upon prior work which tends to be cross-sectional.

Consistent with emerging evidence of the escalation in the rates of food insecurity since the onset of the COVID-19 pandemic (6, 19, 40, 41), we found that the weighted prevalence of food insecurity increased from 4.83% (2018) to 9.54% early in the pandemic (June 2020). While several sociodemographic and socioeconomic characteristics were significantly associated with food security in 2018 and 2020, these associated risk and protective factors appear to have changed early in the COVID-19 pandemic.

With regard to sociodemographic factors, in 2018, non-Hispanic Black participants were more likely to report food insecurity, consistent with other studies finding that race and ethnicity predicted food insecurity among older adult populations (42–44). However, race and ethnicity did not appear to significantly predict food insecurity early in the COVID-19 pandemic. Similarly, those living in rural areas were more likely to report being food insecure in 2018, but not in 2020, in contrast to findings from some other studies that food insecurity worsened among rural populations early in the pandemic (45, 46). The relative shift in factors associated with food insecurity from non-Hispanic Black and rural populations in 2018 to populations of relatively younger age (under 75 years) in 2020 could be attributed to the resilience of rural, non-Hispanic Black, and older adult populations in accessing food during strained and difficult circumstances. It could also speak to the rise in family support, mutual aid societies, and other community-focused strategies to promote food access during the pandemic to rural, older, and Black populations (47). Local community programs such as Meals on Wheels rose to the challenge to address a dramatic surge of demand in food delivery for older adults in urban, suburban, and rural communities, delivering meals to a million more individuals (47% more than pre-pandemic) by July 2020 (48). For Black populations in particular, systemic racism was brought to the forefront in U.S. society during such events as George Floyd's murder in May 2020, which is in the latter portion of the observation period for the present study. For rural populations in particular, increased online access to food and more gardening observed early in the COVID-19 pandemic could have contributed to the attenuated disparities observed in food security experienced by rural populations (45, 49, 50). Further research is needed to assess whether the observed attenuation of disparities in food security for non-Hispanic Black, rural, and relatively older adults continued further into the COVID-19 pandemic and beyond.

With regard to socioeconomic factors, the protective effects of higher levels of education and greater wealth for food insecurity in 2018 are unsurprising. Less intuitive were our findings that education level, wealth, and income were not significant predictors of food insecurity in 2020, contrary to the findings from other studies on the socioeconomic predictors of food insecurity during COVID-19 (21, 22, 26, 35, 51, 52). This finding suggests that the pandemic appears to have affected food security across different socioeconomic strata of middle-aged and older adults, being an equalizer of sorts in that regard. It is also possible that lower-income individuals were aided by pandemic relief such as stimulus funds, mortgage relief, or eviction moratoria. Food security might also reflect other factors related to scarcity (supply chain issues, changes in relative vs. absolute resources) beyond cost alone.

Our findings support that compared to working individuals, older adults who were not working due to a disability, as well as those experiencing greater IADL limitations, experienced significantly higher odds of experiencing food insecurity early in the pandemic. These findings are consistent with prior studies suggesting that people with disabilities are at elevated risk of food insecurity (25, 35, 53–56) and it appears that these risks have become heightened during COVID-19 (57). In a study of Medicare beneficiaries with disabilities, Friedman (56) found that people with one or more disabilities were more likely to be food insecure than non-Medicare beneficiaries during the COVID-19, potentially attributable to fear of going out for food, limited mobility to get food, and barriers accessing food delivery services. Older adults who were not working due to a disability may encounter multiple barriers related to disability and income instability in accessing, procuring, and preparing food items. People with disabilities also incur a substantial number of costs for needed disability-related goods and services, such as for assistive technologies, home care, and medical expenses (58). During times of economic hardship and increasing prices, many people with disabilities may be forced to substitute food security for the purchasing of these needed goods and services. A notable finding from the longitudinal analysis, moreover, is that adults with worsening numbers of IADL limitations and a recent onset of work disability experienced a high risk of food insecurity during the pandemic. This suggests that a greater attention is warranted to preventing food insecurity for older adults experiencing worsening or recently developing functional limitations.

Early in the pandemic, homeownership was also associated with lower odds of experiencing food insecurity in our study. We found that renters, relative to homeowners, were nearly three times more likely to experience food insecurity. The vulnerabilities of renters to food insecurity relative to homeowners have been documented in prior studies before and during the COVID-19 pandemic (21, 51, 59, 60). Individuals and families that struggle with housing instability tend to experience food insecurity (61). Owning a home or having stable, affordable housing might help individuals set aside a larger part of their budget for food and other needed items. Even in the case of income loss, homeownership might provide buffering effects to mitigate the negative consequences of income loss on food security (59). In our sample, the percentage of participants who were homeowners decreased from 79.7% (2018) to 65.9% (2020), while the percentage of participants who were renters nearly doubled from 15.3% (2018) to 30.0% (2020). Those who were homeowners in 2018 but not in 2020 were nearly three times more likely to experience food insecurity. Many circumstances can contribute to pathways from homeownership to renting in older adulthood, including the death of a spouse (62), drops in household income (62), increased costs associated with homeownership (63), and financial shocks (experienced or anticipated) such as housing price changes (64). Therefore, it is likely that some of those who transitioned from homeowners in 2018 to renters in 2020 also experienced other personal and financial stressors that increase their vulnerability in experiencing food insecurity. Furthermore, while selling a home can result in increased wealth liquidity, the high transaction fees associated with selling a home (65) might affect financial—and therefore food—security among older adults. Further investigation of how housing transitions affect food insecurity is needed to identify risk and protective factors for food insecurity among older adults during COVID-19 and other disasters.

## 5. Limitations and directions for future research

Several limitations should also be considered when interpreting the results. Although prior studies have widely utilized the measure of self-reported food insecurity in the HRS (18, 30, 31, 66, 67), the measure is self-reported and has not been validated through more direct measures, such as food intake and expenditures. The self-reported measure may thus not provide an accurate estimation of the prevalence of food insecurity in comparison to these direct measures and thus such validation research is needed in future. The HRS Core interview does not include the USDA Household Food Security Survey, which is commonly utilized as a valid and standardized tool to assess food security status (54). The two-item measure adopted here and available in the HRS is similar to the USDA measure with both relying on self-reported recall of times they could not afford food and reduced desired food intake as a result. Caution is also needed for interpreting the identified changes in food security status as resulting from the COVID-19 pandemic due to challenges in comparing regression coefficients between models (68, 69). Another limitation to inferring causality relates to the imprecision of the measures of food insecurity. For example, in our measure of food insecurity, respondents in 2020 were asked in the past 12 months whether they ever ate less than they felt they should because there was not enough money to buy food. This would include recall of time periods that preceded the pandemic. Though we suspect that there is a contemporaneous bias in how respondents answer such questions that may indicate their food security status in the pandemic, future studies evaluating changes from the pandemic should seek to restrict measures of food insecurity specifically during the period of the COVID-19 pandemic.

As the present study focuses on changes in food security early in the COVID-19 pandemic, future studies should investigate changes in food security throughout the COVID-19 pandemic and beyond. Such research should investigate the impacts of the intensification of drivers of food security observed in the United States and globally on older adults. Such drivers that have further contributed to the high cost and scarcity of nutritious foods—and growing inequalities in nutrition and food insecurity—include continued supply chain problems, economic shocks and growing inflation, conflict, and climate extremes (70). This future research will enhance our understanding of risk factors related to food insecurity over the course of the pandemic and the way in which COVID-related food insecurity influences the long-term health and wellbeing of older adults.

Lastly, due to the limitations of the data, some group sample sizes were small, while others were not examined in the study. The relatively large confidence intervals observed in certain groups (those not working due to a disability, non-Hispanic Other racial/ethnic groups) could be due to the smaller sizes of those groups. While we did find significant differences in food security among these groups (compared to their respective reference groups), the uneven group sizes might have made our findings more conservative. Due to the limitations of race/ethnicity constructs and groupings in the survey design, we were unable to measure the disparate risk of food insecurity during the COVID-19 pandemic among Hispanic/Latinx and Asian subgroups based on countries of origin, as well as among Native American/Alaska Native groups and subgroups. A growing body of literature has revealed that the COVID-19 pandemic has exacerbated food insecurity risk among Native Americans, Asian Americans, and foreign-born Americans (22, 24, 52, 71). These disparities are presumably due to greater social vulnerability to disaster risk resulting from unequal access to resources and already difficult circumstances in the pre-pandemic context (22). Further research is needed to examine the risk and protective factors of food insecurity within these groups.

## 6. Conclusion

The current study highlighted the shift in sociodemographic and socioeconomic predictors of food insecurity among a sample of nationally representative middle-aged and older US adults before and during the early COVID-19 pandemic. COVID-19 food policies and intervention strategies that target older populations should focus more on individuals with disabilities and those vulnerable to economic hardship and housing instability. At a time of social distancing, access to food through the local community and delivery services is critically essential for older individuals. Policies to support local food pantries, food banks, and congregate meal settings—as well as those promoting the stability of neighborhood food supply more generally—can help expand access to community food resources. The increase in accessible and affordable online food shopping and food delivery services would benefit middle-aged and older adults with disabilities and mobility challenges. Older adults may have been particularly socially isolated during the pandemic to minimize the risk of COVID-19 infection. Promotion of more widespread screening for food insecurity and assistance in SNAP enrollment for older adults could be potentially achieved effectively in primary care and home care settings amid the pandemic. Future research is needed to assess the long-term effects of COVID-19 related to food insecurity on the health and wellbeing of older adults. National and community-focused food preparedness strategies targeting groups vulnerable to food insecurity should be further evaluated and supported to prepare for future public health and natural disasters.

## Data availability statement

Publicly available datasets were analyzed in this study. This data can be found here: https://hrs.isr.umich.edu/about.

## Author contributions

EN planned the study, supervised the data analysis, and contributed to writing the manuscript. GJC performed statistical analyses and contributed to writing the manuscript. ZM contributed to the direction of the manuscript, performed statistical analyses, and contributed to writing and revising the manuscript. All authors contributed to the manuscript revision and read and approved the submitted version.

## Footnotes

^1^Assets include the sum of the net values of primary residence, secondary residence, real estate, vehicles, business, all IRA and Keogh accounts, stocks, mutual funds, investment trusts, checking, savings, money market accounts, CD, government savings bonds, T-bills, bonds, and bond funds. Debts include the sum of all mortgages/land contracts (including secondary residence), other home loans (including secondary residence), and other debts.

^2^The objective of this study was to characterize factors that were—and were not—associated with food insecurity before and during COVID-19. Therefore, we opted to include all examined variables in the analyses, whether or not they were found to be significantly associated with food insecurity in descriptive and multivariate analyses. The −2 log-likelihood results were 698.82 for 2018 (*p* < 0.0001) and 1,201.14 (*p* < 0.0001) for 2018.

^3^Preliminary weights are provided that adjust for selection and non-response into the special release. Because 2020 ACS data are not yet available, the post-stratification is only approximate. Respondents born 1966 or later, or who did not give an interview before 2020, do not have weights. The preliminary weight variable is CVWGTR.
